# Age-related appearance of a CMV-specific high-avidity CD8^+ ^T cell clonotype which does not occur in young adults

**DOI:** 10.1186/1742-4933-5-14

**Published:** 2008-11-12

**Authors:** Angelika Schwanninger, Birgit Weinberger, Daniela Weiskopf, Dietmar Herndler-Brandstetter, Stephan Reitinger, Christoph Gassner, Harald Schennach, Walther Parson, Reinhard Würzner, Beatrix Grubeck-Loebenstein

**Affiliations:** 1 Institute for Biomedical Aging Research, Austrian Academy of Sciences, Rennweg 10, 6020 Innsbruck, Austria; 2 Central Institute for Blood Transfusion and Division for Immunology, University Hospital, 6020 Innsbruck, Austria; 3 Institute of Legal Medicine, Innsbruck Medical University, 6020 Innsbruck, Austria; 4 Department of Hygiene, Microbiology and Social Medicine, Innsbruck Medical University, 6020 Innsbruck, Austria

## Abstract

Old age is associated with characteristic changes of the immune system contributing to higher incidence and severity of many infectious diseases. Particularly within the T cell compartment latent infection with human Cytomegalovirus (CMV) is contributing to and accelerating immunosenescence. However, latent CMV infection and reactivation usually does not cause overt symptoms in immunocompetent elderly persons indicating immunological control of disease. Little is still known about the clonal composition of CMV-specific T cell responses in donors of different age. We therefore analyzed CD8^+ ^T cells specific for an immunodominant pp65-derived nonamer-peptide (NLVPMVATV; CMV_NLV_) in different age-groups. Independent of donor age CMV_NLV_-specific CD8^+ ^T cells preferentially use the V beta family 8. This family has monoclonal expansions in the majority of donors after stimulation of CD8^+ ^T cells with the peptide. By sequencing the CDR3 region of the T cell receptor we demonstrated that CMV_NLV_-specific, BV8^+ ^CD8^+ ^T cells share the conserved CDR3-sequence motif SANYGYT in donors of all age groups. Interestingly, a second conserved clonotype with the CDR3-sequence motif SVNEAF appears in middle-aged and elderly donors. This clonotype is absent in young individuals. The age-related clonotype SVNEAF binds to the pMHC-complex with higher avidity than the clonotype SANYGYT, which is predominant in young adults. The dominance of this high avidity clonotype may explain the lack of overt CMV-disease in old age.

## Background

Ageing is associated with an increase in the incidence and severity of many infectious diseases. The most common infections in the elderly are influenza, infections with Streptococcus pneumoniae, infections of the skin and also of the urogenital tract [1]. In addition, reactivation of latent viruses and bacteria such as Varicella-Zoster-Virus leading to Herpes zoster [2,3] and Mycobacterium tuberculosis [4,5] are more frequent in old age. This may be due to decreased immunosurveillance as well as to other factors such as age-associated diseases, poor nutrition, chronic renal failure and institutionalization.

Cytomegalovirus (CMV) is a human beta-herpesvirus with a prevalence of 60–100% in the adult population. The link between CMV-infection, immunosenescence and longevity has recently been a subject of great interest [6,7]. Despite frequent reactivation of latent CMV in the elderly as suggested by increased anti-CMV antibodies and viral shedding in the urine [8] there are no reports about overt CMV-disease in immunocompetent elderly persons. T cells are essential for the control of viral replication, spread and disease [9-12]. In CMV-seropositive elderly persons up to 25% of the total CD8^+ ^T cell pool can be specific for CMV with the epitope NLVPMVATV of the pp65 matrix protein (CMV_NLV_) being immunodominant [13]. These CMV-specific cells show a highly differentiated effector phenotype [14-16] and express markers for cytotoxicity [14,16]. They are proinflammatory [16], and to a high degree clonally expanded [13,17,18]. This has led to the suggestion that CMV-specific T cell clones take up a lot of space and may therefore be responsible for the loss of T cells of other specificities, such as for instance for Epstein-Barr virus (EBV) [19]. The proinflammatory properties of the steadily increasing number of CMV-specific T cells may represent an additional problem, as age-related subclinical inflammatory processes termed "inflamm-ageing" can be enhanced [20]. Inflammation is known to support the development and progression of age-related diseases such as for instance Alzheimer's disease [21]. In longitudinal studies on octo- and nonagenerians CMV-seropositivity has also been linked to the so-called "immune-risk phenotype" and with increased mortality [22,23].

Despite the obvious importance of CMV infection in old age little is known about the clonal composition of CMV-specific T cells in apparently healthy elderly persons. We therefore analyzed the clonal composition of CD8^+ ^T cells, which are specific for the HLA-A*0201-restricted, immunodominant pp65-derived epitope NLVPMVATV [24,25] in persons of different age.

## Results and discussion

### Stimulation of CD8^+ ^T cells with CMV_NLV_-peptide leads to expansion of CMV_NLV_-specific cells with restricted V beta usage

**Figure 1:**
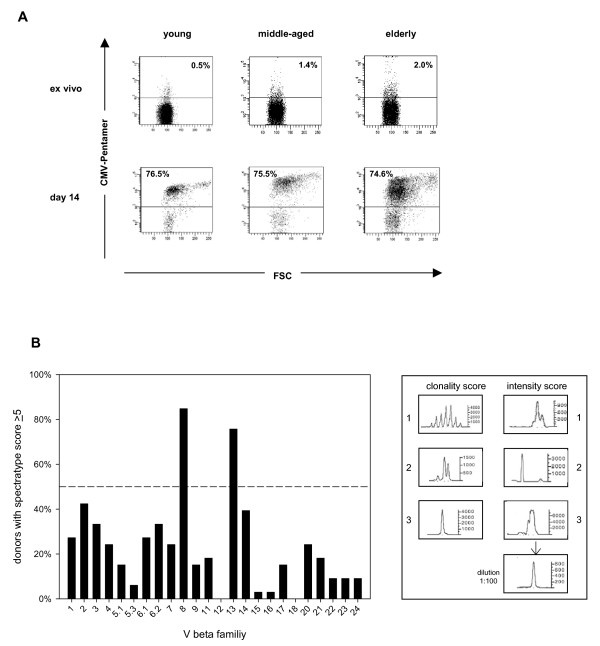
**Preferential expansion of BV8 and BV13 CD8^+ ^T cells after stimulation with CMV_NLV_-peptide**. Cells were stimulated in vitro for 14 days with CMV_NLV_-peptide in the presence of IL-2 and autologous, irradiated PBMC. (A) CD8^+ ^T cells were stained with APC-conjugated pentamers containing the CMV_NLV_-peptide. Representative examples are shown for one young, one middle-aged and one elderly donor directly ex vivo and after 14 days of culture. Percentages of CD8^+ ^CMV_NLV_-specific T cells are indicated. (B) After 14 days of culture CMV_NLV_-specific T cells were further purified from the expanded cells using MACS-technology. Spectratyping was performed from PCR-products of 24 individual V beta families for 31 donors (10 young, 7 middle-aged, 14 elderly). In the right panel examples for the different clonality and intensity scores (see Methods) are shown. Clonality and intensity scores are added to obtain a total score. For each BV family the percentage of donors with a total score above 5 is shown.

CD8^+ ^T cells were isolated from peripheral blood of healthy donors of different age groups and were cultivated for 14 days in the presence of the immunodominant, HLA A*0201-restricted CMV-derived peptide NLVPMVATV, IL-2 and autologous irradiated feeder cells. The frequencies of CMV_NLV_-specific CD8^+ ^T cells were determined ex vivo and were 0.6% ± 0.7, 1.7% ± 0.8, and 1.7 ± 1.9, for young, middle-aged and elderly donors respectively (mean ± SD; p < 0.05 for young vs. middle-aged and young vs. old). Irrespective of age, high frequencies of CMV_NLV_-specific T cells were obtained for most donors after 14 days of culture (mean 77.0% ± 14.9). Figure 1A shows examples of pentamer-FACS-stainings before and after culture for one young, one middle-aged and one elderly donor.

In order to analyze the T cell receptor (TCR) repertoire CMV_NLV_-specific T cells were further purified using pentamers and microbeads reaching a purity of CMV_NLV_-specific CD8^+ ^T cells above 95% for all samples. Spectratyping of the CDR3 (complementary determining region 3) region of the TCR beta chain was performed as previously described [26,27]. CMV_NLV_-specific CD8^+ ^T cells preferentially used BV 8 and BV 13 after culture (Figure 1B). 64% and 59% of all donors had monoclonal expansions (clonality score 3) in BV8 or BV13, respectively. No differences in the spectratype scores were observed between the different age groups (data not shown). Previous work analyzing the V beta usage of CMV_NLV_-specific T cells shows that the broad repertoire of CMV-specific T cells, which is stimulated during primary infection rapidly focuses on individual BV families within the first weeks after infection [28]. Restimulation in vitro does not alter the T cell repertoire [29,30]. In accordance with our results it has been shown that CMV_NLV_-specific T cells preferentially use BV 8, 13 and 6 in healthy adults as well as in immunosuppressed patients [28-33].

### The sequence of the CDR3 region of BV8^+ ^T cell receptors of CMV_NLV_-specific CD8^+ ^T cells changes with age

**Figure 2:**
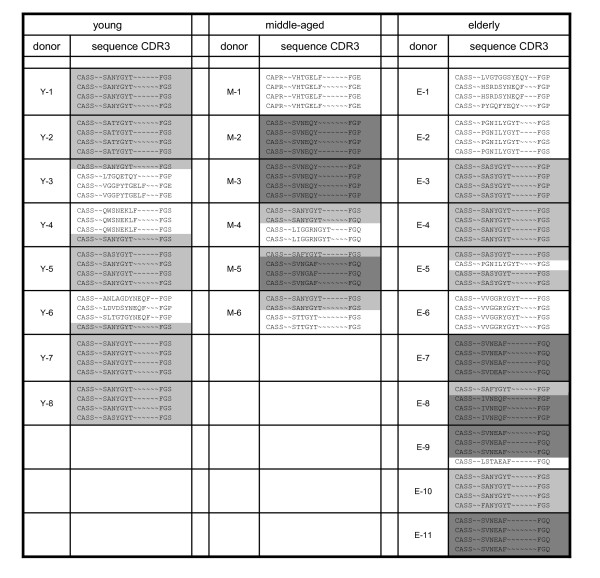
**Sequence analysis of the CDR3-region of BV8^+ ^CMV_NVL_-specific T cells from different age-groups**. PCR-products were generated of the relevant TCR-sequences and cloned into a bacterial vector. 4 arbitrarily chosen bacterial clones are shown for each donor. Two dominant sequences and a variety of individually used sequences were detected. Conserved sequences were categorized as "SANYGYT" (light grey) and "SVNEAF" (dark grey). Discordance of one or two amino acids from the conserved dominant sequences was considered as minor variation, and these clones were included in these two categories. Sequences with greater variations were considered as private clones.

In order to characterize the repertoire of CMV_NLV_-specific CD8^+ ^T cells in more detail we sequenced the CDR3-region of the T cell receptor of in vitro expanded and purified BV8^+ ^T cells. We chose this BV family as the most dominant family within CMV_NLV_-specific T cells. TCR sequences were amplified from cDNA and were cloned into a bacterial vector. Plasmid-DNA was isolated and sequenced by standard procedures. Figure 2 shows the amino acid sequences of the antigen binding site for CMV_NLV_-specific T cell receptors. In accordance with literature on CDR3-sequences from healthy and HIV-infected adults [30-33] the highly conserved CDR3-sequence motif SANYGYT was detected in all young donors. 72% of all bacterial clones derived from young individuals share this sequence motif. 28% of all sequences were private, which means that they were not conserved between different donors. However, we now show that with increasing age the dominance of the clonotype SANYGYT decreases, as this sequence is only present in 21% or 36% of the bacterial clones obtained from middle-aged and elderly donors, respectively. Interestingly, a second dominant clonotype with a shorter CDR3-region and the CDR3-sequence motif SVNEAF appears in middle-aged and elderly donors. Exchanges of one or two aminoacids were considered as minor variations. The age-dependency of CDR3-sequences was analyzed in a cross-table with calculation of Pearson-Chi-square and was shown to be significant (p < 0.01). The CDR3-sequence motif SVNEAF has been detected in previous studies in adult persons [32,33], but was not identified as a conserved sequence. None of this previous work refers to the age of the donors and it can thus be assumed that most of the donors studied were young adults. We now for the first time report that a CMV_NLV_-specific BV8^+ ^clonotype with the CDR3 sequence SVNEAF becomes dominant with increasing age, while the frequency of the "youth related" clonotype SANYGYT decreases.

### CMV-specific BV8+ CD8^+ ^T cells with the TCR-sequence SVNEAF have a higher antigen avidity than corresponding cells with the sequence SANYGYT

**Figure 3:**
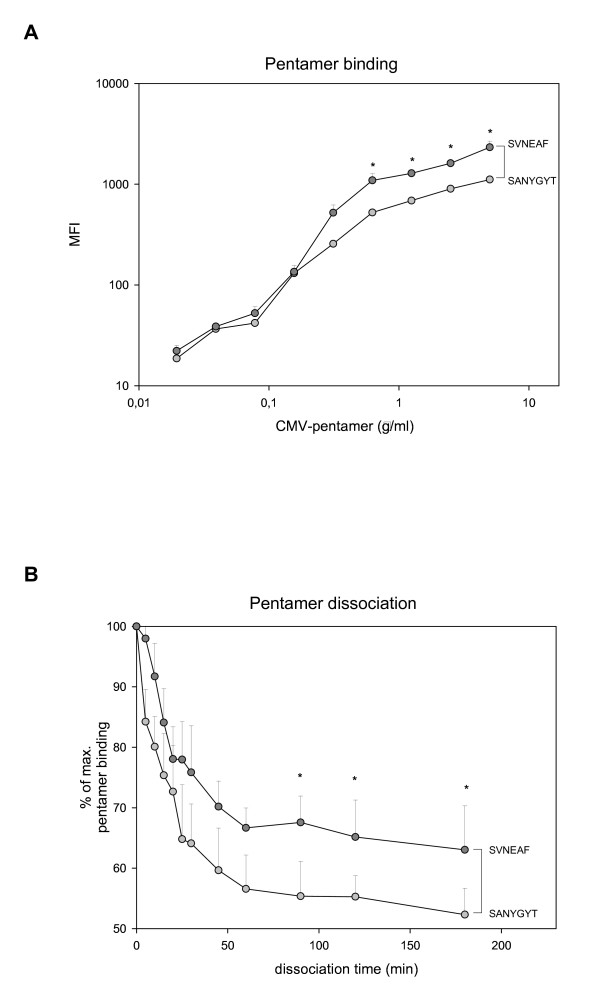
**Higher antigen avidity of CMV-specific CD8^+ ^T cells with the CDR3-sequence SVNEAF**. CMV_NLV_-specific CD8^+ ^T cells were stimulated in vitro with CMV_NLV_-peptide for two weeks. Donors with a monoclonal profile of BV8^+ ^CMV_NLV_-specific T cells with the CDR3-sequence SANYGYT or SVNEAF respectively were selected. BV8^+ ^T cells were identified by FACS-staining and results are shown for BV8^+ ^gated, CMV_NLV_-specific-T cells only. (A) CMV_NLV_-specific BV8^+ ^T cells were stained with different concentrations of CMV_NLV_-pentamer. The mean fluorescence intensity (MFI; note the logarithmic scale) of bound pentamer is indicated for donors with the CDR3-sequence SANYGYT (light grey) or SVNEAF (dark grey). n = 3; mean ± SEM. * *p *< 0.05 (Student's t-test for unpaired data). (B) CMV_NLV_-specific CD8^+ ^T cells were stained with saturated amounts of CMV_NLV_-pentamer (1.25 μg/ml) and the dissociation rates of pentamers from CMV-specific CD8^+ ^T cells were determined for donors with the CDR3-sequence SANYGYT (light grey) or SVNEAF (dark grey). Dissociation of pentamers was assessed by FACS analysis at different time points (range 0–180 min). Mean fluorescence intensity (MFI) of pentamer-positive T cells at time point 0 (maximum pentamer binding) was considered as 100%. n = 3; mean ± SEM. * *p *< 0.05 (Student's t-test for unpaired data).

In order to further investigate CMV_NLV_-specific BV8^+ ^T cells with different conserved CDR3-sequences we analyzed the antigen avidity of T cells with either the CDR3-sequence-motif SANYGYT or SVNEAF. For this purpose cultures were selected, in which BV8^+ ^CMV_NLV_-specific T cells had a monoclonal profile with the CDR3-sequence SANYGYT or SVNEAF, respectively. Binding and dissociation of pMHC pentamers to the T cell receptor of in vitro expanded BV8^+ ^CMV_NLV_-specific T cells were analyzed. Staining with increasing amounts of CMV_NLV_-pentamer shows that pentamer binding and therefore avidity of the TCR is significantly increased for T cells with the CDR3-sequence SVNEAF compared to corresponding cells with the CDR3-sequence SANYGYT (Figure 3A; note the logarithmic scale). The kinetics of TCR-pMHC dissociation provides information on the stability of the TCR-pMHC complexes. T cells were stained with saturated amounts of CMV_NLV_-pentamer and anti-HLA-A2*0201 antibodies were added to prevent re-binding of dissociated pentamers. We could show that CMV-pentamers dissociated more slowly from CMV-specific CD8^+ ^T cells with the CDR3-sequence SVNEAF, whereas TCR-pMHC complexes on cells with the sequence SANYGYT were less stable (Figure 3B). In summary these data show that the interaction between pMHC complexes and the TCR is stronger and more stable for T cells that express a TCR with the antigen-binding-motif SVNEAF indicating higher avidity of these T cells.

High avidity CD8^+ ^T cells are essential for clearance of viral infections [34-37] and for the elimination of tumour cells [38-40]. Therefore, anti-tumour vaccination aims to induce high avidity tumour-specific T cells. In concordance, immunotherapy for viral diseases such as EBV- or CMV-reactivation and infection in immunosuppressed patients after haematopoietic stem cell transplantation benefits from selection of virus-specific T cells with high avidity [41]. Regarding our data, the question arises, why the high avidity clonotype SVNEAF is absent in young individuals while it is dominant in a subset of the middle-aged and elderly persons. In view of the well known decline of immune function with age [42] it seems likely that a subgroup of elderly persons suffers from high CMV-activity and pronounced viral reactivation [8], which necessitated the expansion of the high avidity BV8^+ ^CMV_NLV_-specific clonotype for viral control. Due to its high avidity this clonotype expands and presumably exerts increased effector functions in vivo leading to protection from CMV-disease. However, accumulation of CMV-specific T cells of this specific clonotype may be particularly prone to inhibit T cells of other specificities [19]. CMV-seropositivity has also been associated with non-responsiveness to anti-influenza vaccination [43] and with frailty [44]. The price for good protection against CMV -as provided by CMV-specific cytotoxic T lyphocytes with high avidity- might thus be accelerated immunosenescence and potentially lower responses to other pathogens.

## Methods

### Blood donors

Peripheral blood was obtained from healthy, CMV-seropositive, HLA A*0201- positive donors of different age groups (Table 1). A medical history was obtained and only individuals without malignancies, acute diseases or advanced stages of severe chronic diseases, such as chronic inflammatory disease, atherosclerotic disease, congestive heart failure, poorly controlled diabetes mellitus, renal or hepatic disease or chronic obstructive pulmonary disease and persons without immunosuppressive therapy were included in the study. The study was approved by the local ethical committee and all participants gave their written informed consent. Peripheral blood mononuclear cells (PBMC) were isolated by density gradient centrifugation (FicollHypac; Amersham Biosciences). CMV-specific antibody levels were determined by enzyme-linked immunosorbent assay (Enzygnost^® ^anti-CMV/immunoglobulin G; Dade Behring) according to the manufacturer's protocol. Recent primary infection with CMV is associated with the presence of low-avidity CMV-specific antibodies. In a fluorescence-based assay (Euroimmun) the avidity of CMV-specific IgG was measured by analyzing the stability of the antigen-antibody-complex in the presence of urea. Low-avidity antibodies show a "relative avidity index" (RAI; optical density in the presence of urea compared to optical density without urea) of <40%. For all samples tested the RAI was >45% indicating past infection and excluding recent primary infection.

**Table 1:** Gender and age of blood donors

	n	male/female	median age (years)	age range (years)
young (≤ 39 y)	10	4/6	33	28–37
middle-aged (40–64 y)	7	3/4	49	42–53
elderly (≥ 65 y)	15	7/8	70	65–87

### Cultivation of CD8^+ ^T lymphocytes and enrichment of CMV_NLV_-specific T cells

CD8^+ ^T cells were isolated using anti-CD8-conjugated microbeads and the magnetic-activated cell sorting (MACS) system (Milteny Biotech) according to the specifications given by the manufacturer. CD8^+ ^T cells were cultivated in RPMI 1640 (Cambrex) supplemented with 10% FCS (Sigma-Aldrich) and 1% penicillin-streptamycin (Cambrex) at 37°C and 5% CO_2_. T cells were stimulated for 14 days with 0.1 μg/ml of the immunodominant peptide NLVPMVATV (Bachem) derived from the CMV-encoded protein pp65 in the presence of IL-2 (20 ng/ml) and irradiated (30 Gy) autologous PBMC in a CD8^+ ^: PBMC ratio of 1:1. IL-2 (20 ng/ml) was added every three days and cells were restimulated with peptide and irradiated autologous PBMC after 7 days. Percentages of CMV_NLV_-specific CD8^+ ^T cells were determined prior to culture and after 14 days of stimulation by immunofluorescence surface staining with APC-coupled pentamers containing the CMV_NLV _peptide (Pro5^® ^MHC, Proimmune). After 14 days of cultivation CMV_NLV_-specific CD8^+ ^T cells were purified using APC-conjugated CMV-pentamer, anti-APC-antibodies coupled with magnetic beads and MACS-technology. Purity of CMV_NLV_-specific T cells was >95% in all cases.

### Isolation of RNA and cDNA synthesis

RNA was isolated from CMV_NLV_-specific T cells using the RNeasy Plus Mini Kit (QIAGEN) and cDNA-synthesis was performed using a Reverse Transcription system with Oligo(dT)-primers (Promega).

### CDR3 spectratyping of V beta families

PCR fragments were amplified from cDNA for 24 V beta families (BV) and complementarity determining region (CDR3) spectratyping was performed as previously described [26,27]. Analysis of the raw data was performed with the GeneScan 3.7 analysis software package (PE Applied Biosystems) using the Local Southern method for fragment size estimation [27]. The occurrence of dominant clonal expansions was quantified for each V beta family by assigning scores for clonality (1 = Gaussian distribution; 2 = several peaks; 3 = one peak; compare with [28]) and intensity as measured in relative fluorescence units (RFU) (0 = < 500 RFU; 1 = 500–3000 RFU; 2 = 3000–8000; 3 = >8000). If necessary, PCR-products were diluted prior to spectratyping and the dilution factor was taken into account for the calculation of the intensity score. Diversity and intensity scores were added and BV families with a total score of ≥ 5 were considered as predominant. This score equally weights monoclonal BV families with intermediate intensity (3+2) as well as oligoclonal BV families with high intensity (2+3). Both categories as well as monoclonal BV families with high intensity (3+3) were considered as predominant.

### Bacterial cloning of TCR-sequences and sequence analysis

T cell receptor sequences of the BV8 family were amplified using a forward primer specific for the BV8 family (5' CGTTCCGATAGATGATTCAGG 3') and a reverse primer (5' CTGGGTCCACTCGTCATTCT 3') located in the constant region of the TCR beta chain. PCR fragments were cloned into the pCR^®^-II-TOPO^® ^vector (Invitrogen) via TA-cloning and the vector was transformed into chemically competent E. coli (One Shot TOP10, Invitrogen). For each donor several positive clones were picked and plasmid DNA was extracted using standard procedures (QIAprep Spin Miniprep Kit, QIAGEN). TCR-sequences were determined by standard sequencing procedures (QIAGEN) and sequence data were analyzed using the Bioedit Sequence Alignment Editor 7.0.5.3.

### Measurement of TCR binding kinetics

CMV_NLV_-specific CD8^+ ^T cells were harvested after 14 days of culture and used for pentamer binding and dissociation assays as previously described [29,30] with minor modifications. Briefly, T cells were stained with increasing amounts (range 0.1 to 5 μg/ml) of CMV_NLV_-pentamers. Mean fluorescence intensity (MFI) of pentamer-binding CD8^+ ^T cells was measured by a FACSCalibur flow cytometer (BD Pharmingen). For the analysis of pentamer dissociation T cells were stained with saturated amounts of CMV_NLV_-pentamers (1.25 μg/ml). 25 μg/ml unlabelled anti-HLA-A2*0201 antibodies (clone BB7.2) were added to prevent re-binding of dissociated pentamers. Dissociation of pentamers was assessed by FACS analysis at different time points (range 0–180 min). The MFI of pentamer-positive T cells at time point 0 (maximum pentamer binding) was considered as 100%.

## Competing interests

The authors declare that they have no competing interests.

## Authors' contributions

AS carried out the TCR avidity studies. BW carried out cloning and sequencing experiments and prepared the manuscript. DW carried out cloning and sequencing experiments. DHB performed FACS-analysis. SR participated in the analysis of sequence data. CG collected blood from healthy donors and performed HLA-analysis. HS collected blood from healthy donors and performed HLA-analysis. WP performed the spectratyping. RW performed antibody analysis. BGL designed the study and supervised the preparation the manuscript.
